# Transcription Factors behind *MYB98* Regulation: What Does the Discovery of *SaeM* Suggest?

**DOI:** 10.3390/plants13071007

**Published:** 2024-03-31

**Authors:** Prakash B. Adhikari, Xiaoyan Liu, Chen Huang, Nobutaka Mitsuda, Michitaka Notaguchi, Ryushiro Dora Kasahara

**Affiliations:** 1 Biotechnology and Bioscience Research Center, Nagoya University, Nagoya 464-8601, Japan; notaguchi.michitaka.c4@f.mail.nagoya-u.ac.jp; 2 College of Life Science, Fujian Agriculture and Forestry University, Fuzhou 350002, China; liuxy@fafu.edu.cn (X.L.); huangchen0502@163.com (C.H.); 3 Bioproduction Research Institute, National Institute of Advanced Industrial Science and Technology (AIST), Tsukuba 305-8560, Japan; nobutaka.mitsuda@aist.go.jp

**Keywords:** *MYB98*, *SaeM*, regulatory workflow, GT-1, transcription factor

## Abstract

*MYB98* is master regulator of the molecular network involved in pollen tube attraction. Until recently, it was unclear how this gene exhibits exclusively synergid cell-specific expression in ovule. Our recent study has established that a 16-bp-long *SaeM* element is crucial for its synergid cell-specific expression in ovule, and an 84-bp-long fragment harboring *SaeM* is sufficient to drive the process. In this study, we have developed a workflow to predict functional roles of potential transcription factors (TFs) putatively binding to the promoter region, taking *MYB98* promoter as a test subject. After sequential assessment of co-expression pattern, network analysis, and potential master regulator identification, we have proposed a multi-TF model for *MYB98* regulation. Our study suggests that ANL2, GT-1, and their respective homologs could be direct regulators of *MYB98* and indicates that TCP15, TCP16, FRS9, and HB34 are likely master regulators of the majority of the TFs involved in its regulation. Comprehensive studies in the future are expected to offer more insights into such propositions. Developed workflow can be used while designing similar regulome-related studies for any other species and genes.

## 1. Introduction

Gene regulation is tightly regulated in living organisms. Technological advancements and availability of a broader range of data in recent decades have allowed researchers to develop and evolve better if not novel tools and methods to make data-based hypotheses and relatively sound experimental designs. These tools and methods are expected to evolve and change with the advancement of techniques and expansion of available data resources. In this manuscript, we have prepared a simple workflow to formulate hypotheses on potential functional roles of transcription factors (TFs), taking yeast-one-hybridization (Y1H) assay-derived data as input. Although the workflow could be equally applicable to other species, the data analyzed in this study are from Arabidopsis unless stated otherwise. We used the developed workflow to assess potential roles of TFs behind *MYB98*, a gene encoding R2R3 MYB TF.

*MYB98* is pivotal in regulating pollen tube attraction towards the ovule, a critical step in plant pollination and fertilization [1,2]. Synthesis of molecules, particularly cysteine-rich proteins directly involved in pollen tube attraction, depends on exclusive expression of *MYB98* at synergid cells (SCs), the accessory but crucial female gametophyte component cells [3]. *MYB98* is essential for proper filiform apparatus formation at the micropylar end of SC [2,3]. The functional state of this component cell is essential for secretion of pollen tube attractant molecules at the micropyle [3]. Most such molecular players act downstream of *MYB98* and have long been discovered and studied [4,5,6]. However, the molecular players and the mechanism underlying the SC-specific expression of *MYB98* itself had remained obscure until recently. Studies have reported that some TFs like central cell-specific *AGL80* and *CCG* regulate *MYB98* expression. The former represses *MYB98* in the central cell [7], while the latter enhances its expression [8]. However, the mechanism behind the involvement of central cell-specific TFs in SC-specific *MYB98* expression has largely remained a mystery. Moreover, no such regulators have been reported in the SC itself even almost two decades after *MYB98* discovery [2].

Recently, we showed that *MYB98* promoter (*pMYB98*) harbors a cis-element crucially important for its SC-specific expression [1]. Mutation within this 16 bp element, named *SaeM* (*S*C-specific *a*ctivation *e*lement of *MYB98*), essentially led to complete loss of its SC-specific activation. We additionally demonstrated that an 84 bp *pMYB98* cis-region harboring *SaeM* in the middle is sufficient to drive such exclusive expression. We showed that a homeodomain protein, ANL2, and its paralogs HDG1, ETD1, and HDG7 constitute binding potential to *SaeM* [1]. Studies have shown and suggested that crediting a specific expression pattern of a gene to particular TF might be misleading, as such a pattern could be the result of physical association and/or interaction among additional DNA-binding and non-binding TFs [9,10,11,12]. TFs’ interaction with each other brings changes to their structural configurations, thereby affecting DNA the binding efficiency of a thus-formed TF complex [13]. Here we have developed a workflow to sequentially carry out co-expression analysis, interactome analysis, and master regulator prediction for a gene-of-interest, and used it to propose a potential regulatory model behind spatial and temporal expression of *MYB98*.

## 2. Results

### 2.1. Transcription Factor Pool and Analysis Workflow Preparation

**Figure 1:**
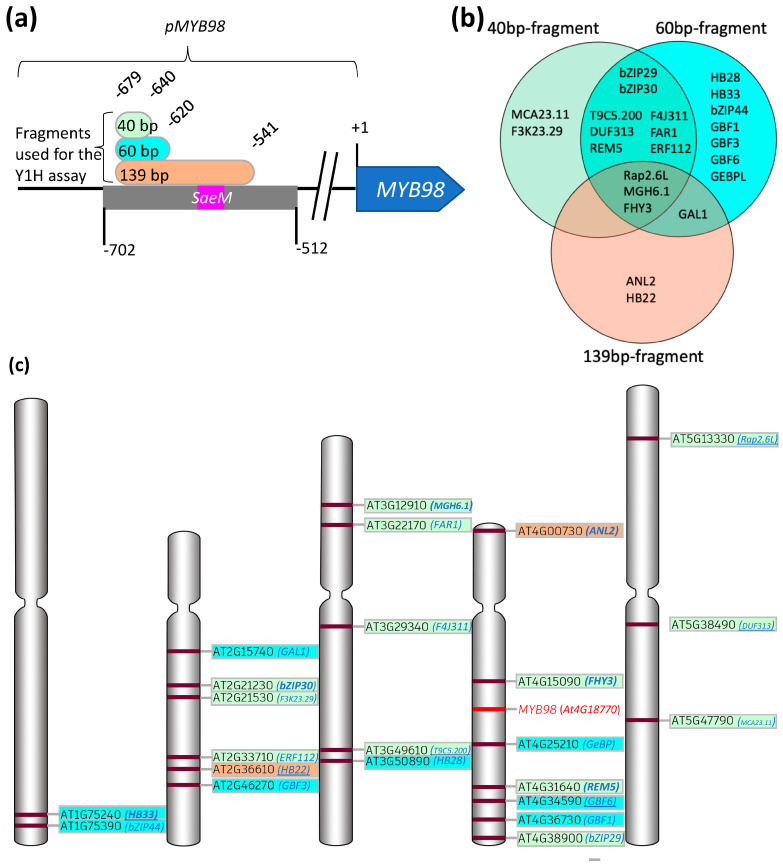
Identification of transcription factors (TFs) with binding affinity to *pMYB98*. (**a**) Three *pMYB98* fragments (40 bp, 60 bp, and 139 bp) used in yeast-one-hybridization (Y1H) assay. Relative positions of functionally active 191 bp region and *SaeM* [1] are marked in gray and red, respectively. (**b**) TFs derived from Y1H assay and their association with *pMYB98* fragments used for this study. (**c**) Chromosomal position of Y1H-assay derived TFs. Members exhibiting binding affinity to all or at least to the shortest fragment (40 bp; −679 to −640 *pMYB98* region) are highlighted in light green; those with affinity unique to the longest fragment (139 bp; suggestive binding site −620 to −541 bp *pMYB98* region) are in orange; and the remaining TFs showing affinity uniquely to 60 bp fragment or both 60 bp and 139 bp fragment (suggestive binding site −640 to −620 bp *pMYB98* region) are in cyan. *MYB98* (in red) is included for reference.

The *SaeM* element is positioned in the middle of a 191-bp-long functionally active *pMYB98* region (Figure 1a). Among 23 TFs derived from our yeast-one-hybridization (Y1H) assay carried out using fragments within this region, the majority localize to chr2 and chr4 (Figure 1b,c), two Arabidopsis chromosomes sharing high levels of similarity [14,15]. The proximity of TFs to their target gene is linked to their efficiency in finding a binding site in the target genes’ promoter as suggested by a study of prokaryotes [16]. Whether a similar case applied to *MYB98* regulators in the distant past is likely but remains unclear.

**Figure 2:**
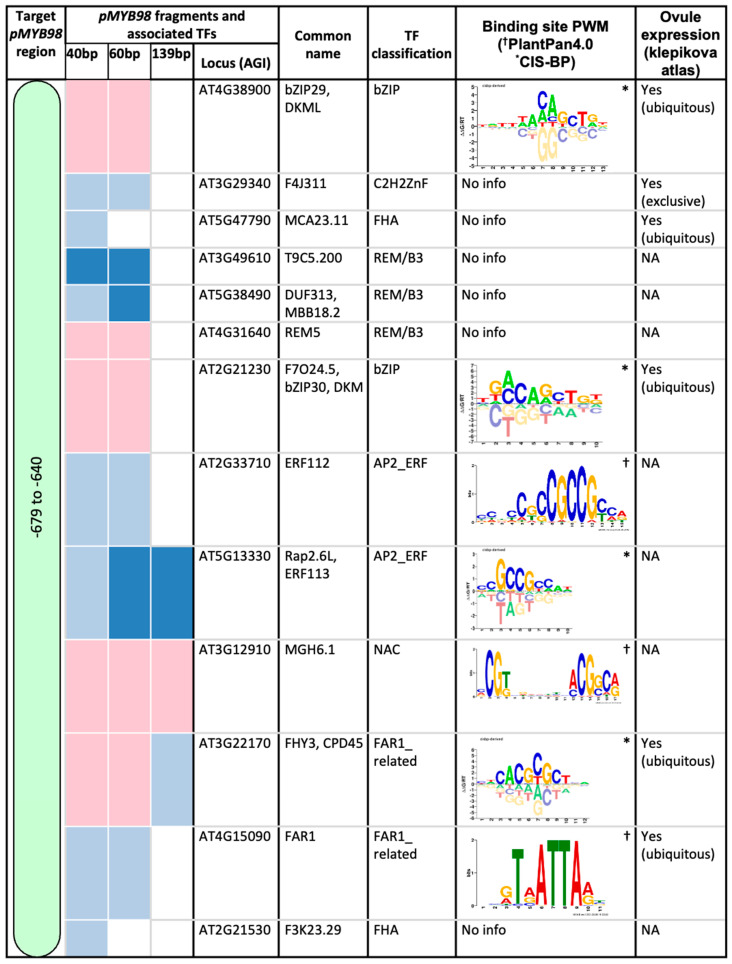

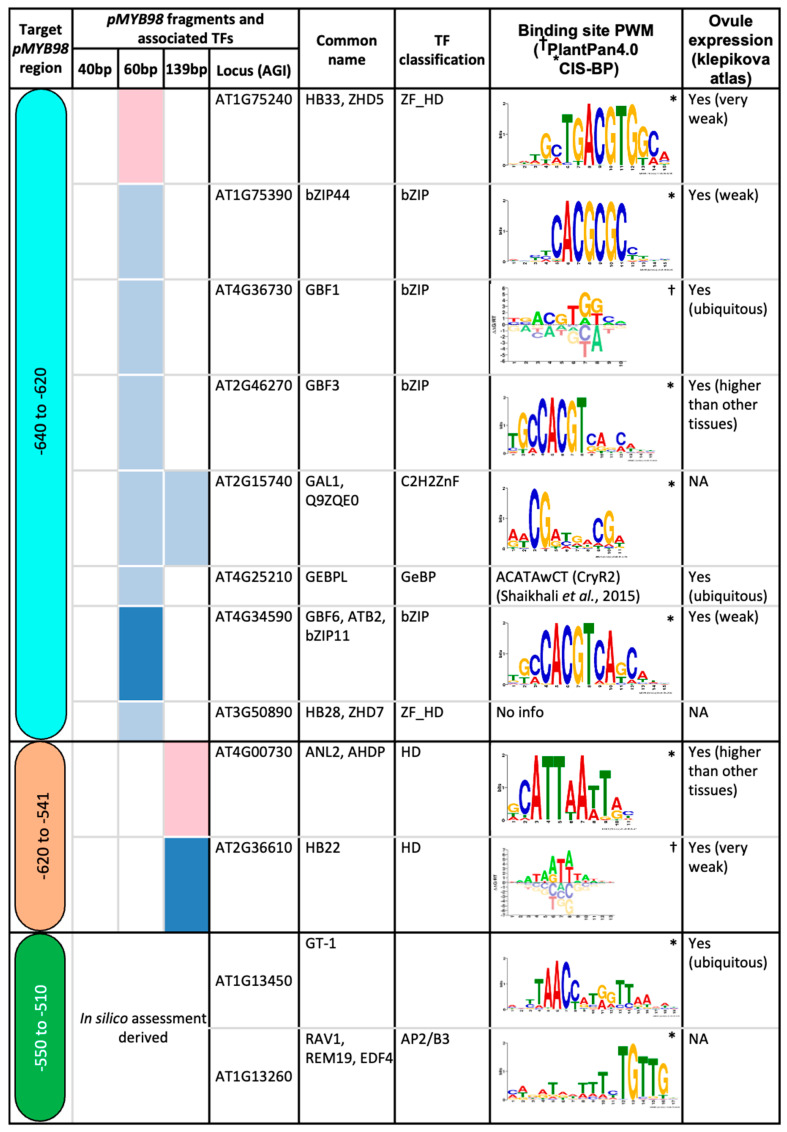
Transcription factors (TFs) derived from Y1H experiment. Far-left column indicates potential *pMYB98* regions targeted by associated TFs. Color coding in subsequent columns denotes the strengths of TF-binding affinity to respective *pMYB98* fragments used in Y1H experiment (red = strong; dark blue = weak; light blue = very weak) (refer to Figure 1a). † PlantPan4.0 [19]-derived, * CIS-BP [20]-derived; ovule expression data were derived from the klepikova atlas [21].

**Figure 3:**
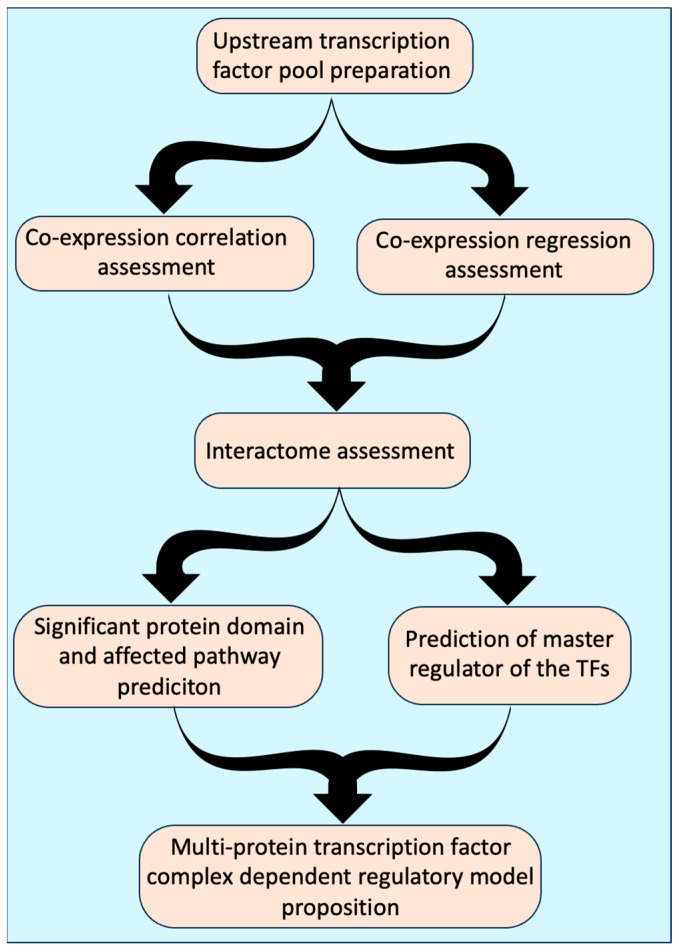
Workflow of regulome assessment and regulatory complex prediction for *MYB98*.

Several of the Y1H assay-derived TFs were common across all *pMYB98* sub-fragments used in current study (Figure 1b and Figure 2), suggesting direct or indirect association with the shortest fragment. This group includes TFs with REM/B3 (3), bZIP (2), AP2/ERF (2), FHA (2), FAR-related (2), NAC (1), and C2H2 zinc finger (1) domains. Only one TF (GAL1) was common between the TF pools of 60 bp and 139 bp fragments (Figure 1b and Figure 2). TFs unique to the 60-bp-derived pool or present in both the 60 bp and 139 bp pools are likely associated with the 3′-region of the 60 bp fragment not overlapping with its shorter 40 bp counterpart, i.e., −640 bp to −620 bp. This subset includes TFs with bZIP (4), ZF_HD (2), GeBP (1), and C2H2 zinc finger (1) domains. Among the three, the 139-bp-derived pool contains the smallest number of TFs (6 in total), with only two (both with HD domain) unique to it as compared to other fragment-derived pools, suggesting association with the 3′-region of the 139 bp fragment not overlapping with other fragments, i.e., −620 to −541 bp (Figure 1b and Figure 2). While some studies suggest repressive or transactivation roles for some of the Y1H assay-derived TFs, using them as a sole measure to predict TFs’ role in *MYB98* regulation seemed a little far-fetched. Hence, we developed a simple workflow for this purpose, considering co-expression and potential physical associations of TFs while predicting their functional roles (Figure 3).

In the in vivo reporter expression assay, we observed that the latter region of the functionally active 191 bp *pMYB98* region confers a repressive effect [1]. Since this fragment was not used in the Y1H assay, we checked available DNA affinity purification sequencing (DAP-Seq) data for potential TFs binding within this region (i.e., −550 bp to −510 bp). This region harbors a GT1CONSENSUS cis-element (GRWAAW) [17] in reverse orientation and potential RAV1 binding region (CAACAaCACCTa) (Figure 2) which reportedly binds by recognizing a bipartite DNA sequence composed of CAACA and CACCTG CACCTG as reported in Arabidopsis [18]. Interestingly, an additional potential GT-1 recognition motif overlaps with the *SaeM* 5′ region [1]. Analysis of DAP-seq data suggested potential TFs binding within the functionally active 191 bp promoter fragment including members with WRKY (9), ZF-HD (3), and HD (2) domains. TFs from all the aforementioned analyses were combined into a single pool (38 TFs in total). Based on an earlier assessment [1], we included TOPLESS (TPL), an EAR-motif binding TF with potential to interact with ANL2 and its homologs as well as MYB98 itself (as positive control), AGL80 (as negative control), and a random MYB (MYB63) in the TF pool (42 TFs in total) for downstream analysis following the aforementioned workflow (Figure 3).

### 2.2. Co-Expression Potential: An Initial Clue to TF Functionality

**Figure 4:**
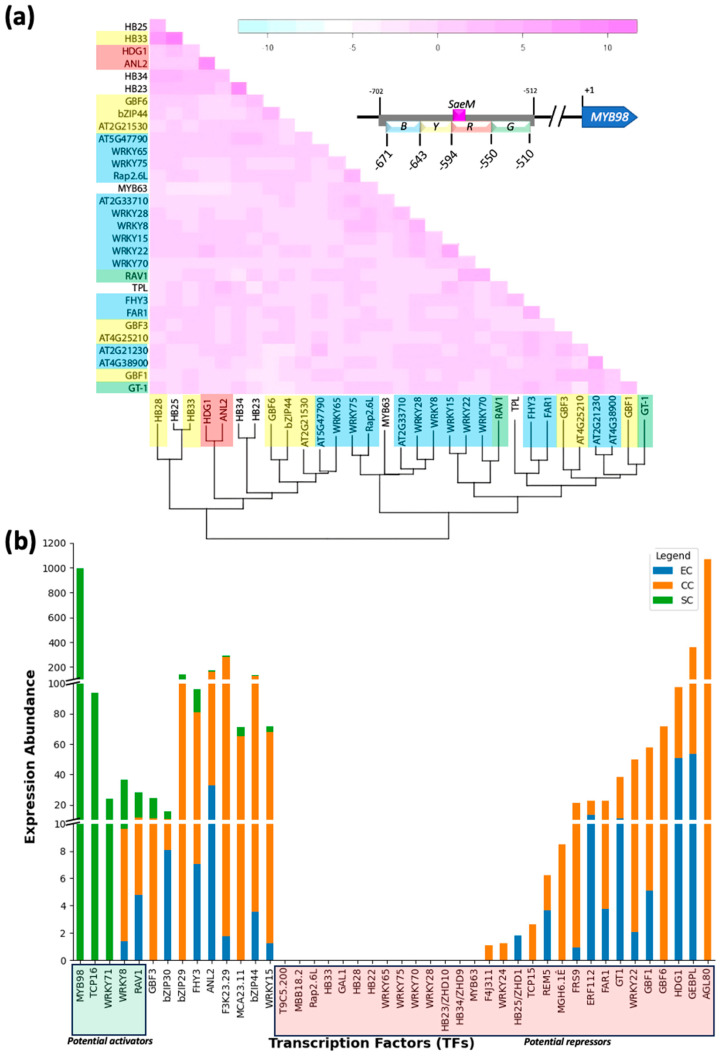
Assessment of co-occurrence and binding potentials of transcription factors (TFs) with putative binding affinities to *pMYB98*. Co-expression hierarchical clustering of TFs. (**a**) Clustering was performed using Ward method at ATTED-II database [19]. Relative co-expression strengths are displayed in color range from cyan to pink (weakest to strongest). Highlight color of TFs indicates their putative binding region within *pMYB98* (B, Y, R, and G). Relative positions of *pMYB98* sub-fragments are displayed in inset. Correlation/regression plots for co-expression of majority of putative TFs with *MYB98* are presented in Figure S1. (**b**) Expression abundance of putative TFs related to *MYB98* regulation in micro-tissues (independent female gametophyte component cells; Arabidopsis) is shown. TFs with no expression in SCs are marked as potential repressors, while those with significantly higher expression at SCs are marked as potential activators (Data source: Song, et al. [23]).

Our initial assumption posited that the co-expression potential of TFs might indicate their stimulatory or antagonistic effects. The ATTED-II database [22] was utilized for in silico analysis. It revealed that at least 30 TFs exhibit varying levels of co-expression potential (Figure 4a). Notably, however, *MYB98* and *AGL80* were missing from the co-expression matrix. Moreover, none of the TFs were among the top 2000 *MYB98* co-expressed genes retrieved from the eFP browser [21]. Consequently, we examined the expression correlation of each TF with *MYB98* individually. Although none of the TFs had an *R*^2^-value near 0.5, they showed slightly positive or negative correlation with *MYB98* (Figure S1).

It is generally accepted that positive and negative correlations can imply respective stimulatory and inhibitory roles of TFs to their target gene; precise conclusions often depend on data standardization and narrowing down to the tissue level. In the absence of such micro-tissue-level expression data, co-expression and correlation/regression analyses so far merely suggest that TFs with a positive expression-correlation to *MYB98* are at least expressed in ovules. Yet a TF could act as a repressor if expressed in *MYB98* non-domain (non-SC) ovule tissues. *AGL80*, a known repressor of *MYB98* in the central cell of the ovule [7], is one such example, exhibiting the highest positive expression correlation to *MYB98* (Figure S1).

Assuming that micro-tissue-level single-cell expression data might yield more reliable insights, we evaluated the expression levels of TFs in FG component cells (data from Song, et al. [23]). The results demonstrated that *AGL80* and *MYB98* are not co-expressed; the former is exclusively expressed in the central cell, while the latter is specifically expressed in SCs (Figure 4b), aligning with previous findings by Zhang et al. [7]. The plot indicates that TF genes like *TCP16* and *WRKY71* show *MYB98*-like SC-specific expression, while roughly 30 other TFs analyzed show little to no expression at SC (Figure 4b), suggesting their potential negative role, if any, in *MYB98* expression.

### 2.3. Network Analysis and Potential Physical Association Determination

**Figure 5:**
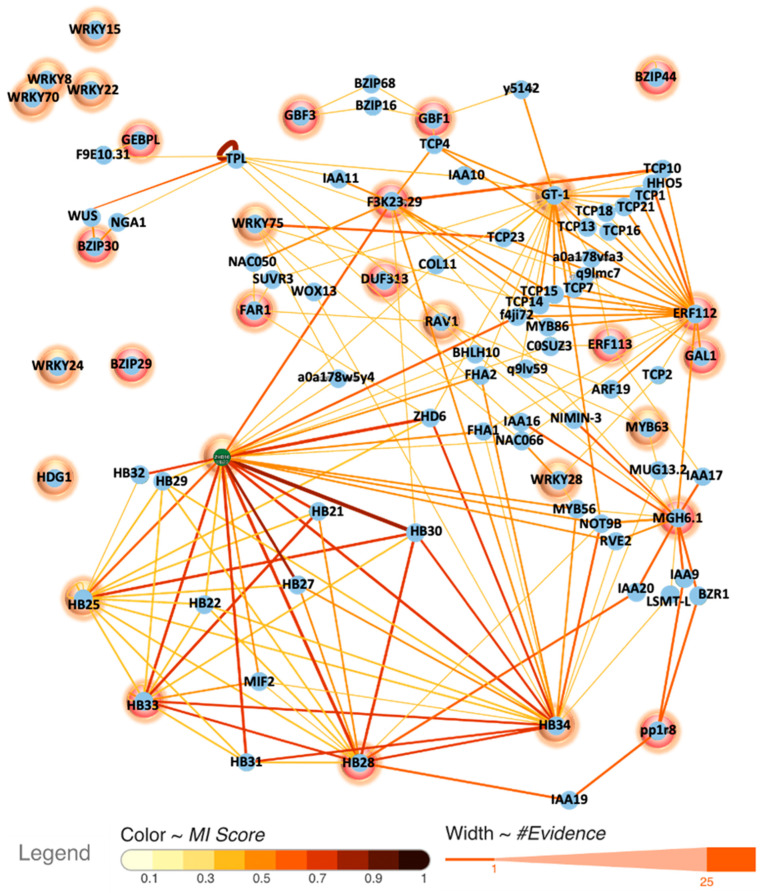
Interaction network of *pMYB98* binding TFs (Y1H-derived circled in red and others in orange). Additional TFs with direct physical association are displayed. TPL is included to illustrate its potential interacting partners in network. The network was generated using IntAct [24].

**Figure 6:**
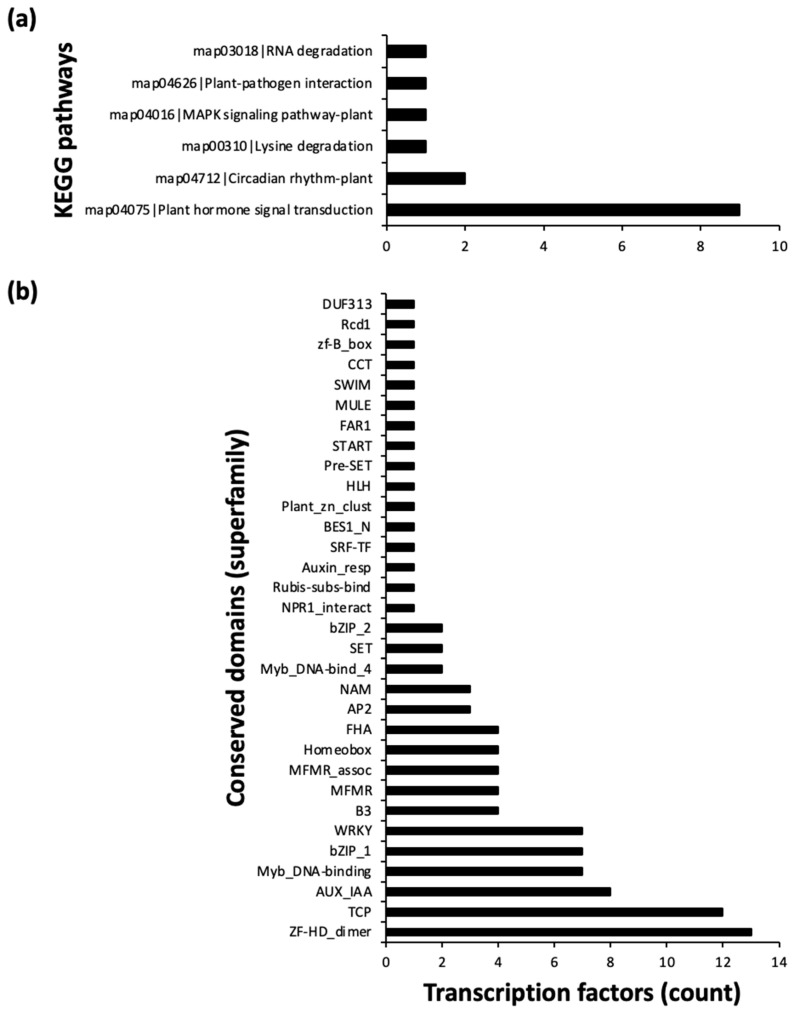
Assessment of potential TFs with physical association with *pMYB98* or putative TFs binding to it (84 in total). (**a**) KEGG pathway analysis of TFs shows 9 belong to hormone signal transduction pathway. (**b**) Conserved domain analysis of TFs reveals two domains (zinc finger HD_dimer and TCP) are the most prevalent (≥10). Note that not all TFs possess defined conserved domains and not all were assigned to a pathway.

Through Y1H assays and in silico analysis, we identified a group of TFs potentially central to *MYB98* regulation, and the co-expression assessment suggested their possible functional roles. However, fragments used for the Y1H assay do not encompass all sites recognized by TFs in the pool (Figure 2). Therefore, we postulated involvement of intermediary TFs in facilitating their interaction with *pMYB98*. Under this premise, we conducted network analysis to identify potentially associated intermediary TFs using IntAct [24] (Figure 5). Since some of the TFs in the initial pool, such as ANL2 and HDG1, possess EAR motifs, we included TPL, a known EAR-dependent gene regulation mediator [25,26], in the network assessment. It predicted potential intermediary partners with physical associations between TFs. The complete network also reveals additional TFs potentially physically associated with the initial pool TFs (Figure S2). The majority of TFs in the network shown in Figure 5 are associated with plant hormone signal transduction, with ZH-HD_dimer and TCP domains being the most prevalent (Figure 6).

**Figure 7:**
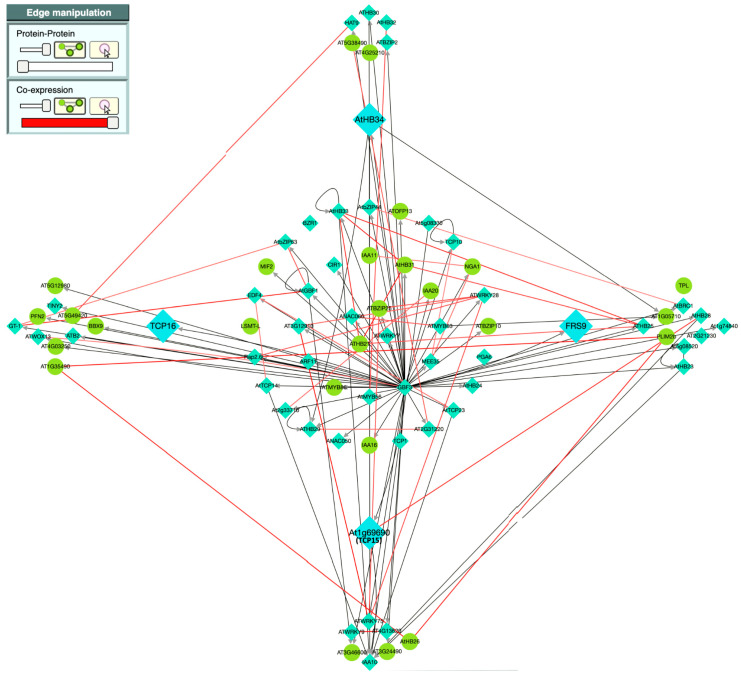
Putative master-regulators of majority of upstream transcription factors of *MYB98* prediction via TF2Network [27]. Transcription factors and other genes are represented by blue diamonds and green circles, respectively. Each black arrow indicates a regulator (at base) and target (at tip); putatively co-expressed genes/TFs are linked with red lines.

Downstream assessment of network-analysis-derived intermediary TFs containing pool using TF2Network [27] suggests that four TFs—TCP15, TCP16, FRS9, and HB34—potentially act as master regulators involved in the regulation of the majority of the TFs in the pool (Figure 7).

## 3. Discussion

### 3.1. Workflow-Dependent Functional Role Assignment to Putative Upstream TFs

Predictions made in this study are partially supported by earlier experimental studies. Examples include *AGL80*’s repressive role on *MYB98* [7]; *WRKY71*’s transactivation role on genes like *RAX*, *FT*, and *LFY* [28,29]; and the antagonistic role of *GeBP*/*GeBPL* on negative regulators of *ARR* genes [30]. Since a transactivator of one gene can have a repressive effect on others, targeted studies are essential to confirm roles of the mentioned TFs on *MYB98* specifically. Nevertheless, a general understanding of potential functional roles of putative upstream TFs should aid in designing further studies. Our study shows that tissue-specific and single-cell expression data offer more precise predictions; evaluating multi-time-point expression data could greatly improve prediction reliability in the temporal dimension as well. This approach would allow screening of genes conferring positive effects from non-domain tissues of the gene-of-target. According to the study by Li et al. [8], such TF genes for *MYB98* include *CCG* and *CBP1*. They are expressed in the central cell, and a defect of at least *CCG* leads to reduced *MYB98* and its downstream gene expression at SCs.

### 3.2. Potential Multi-TF Regulatory Model of MYB98 Expression

**Figure 8:**
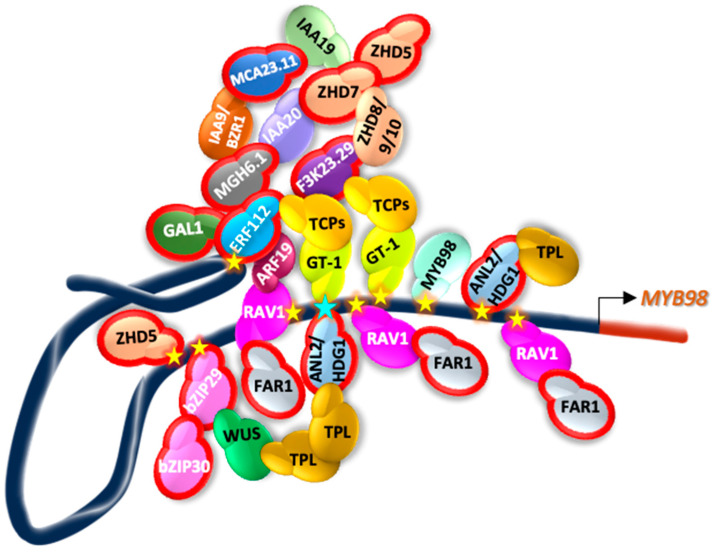
Proposed model for MYB98 regulation. TFs potentially binding to *pMYB98* as indicated by Y1H assay, DAP-seq data, and IntAct analyses. TFs outlined in red were identified through Y1H. The figure illustrates potential physical associations and likely interactions among TFs. Stars mark TF-to-*pMYB98* interaction sites, with *SaeM* indicated by a cyan star. Locations of putative TF binding on *pMYB98* were determined from available DAP-seq and secondary data sources.

Overall analyses suggest that some TFs may bind directly to the *pMYB98* region used for the Y1H assay, while others could regulate *MYB98* expression indirectly. In conclusion, we propose a multi-TF regulatory model, which includes potential TFs directly bound to *pMYB98* (ZHD5, RAV1, bZIP29, ANL2/HDG1, GT-1, MYB98, and ERF112), and those may physically associate to them (Figure 8). This model suggests direct or indirect involvement of TFs in *MYB98* regulation. Temporal and spatial expression of *MYB98* is likely influenced by the interplay between these repressors and transactivators. However, comprehensive experimental data are needed to draw such a conclusion.

### 3.3. Potential TFs on Conferring Tissue Specificity to MYB98

Our earlier study showed that *SaeM* is crucial and the 84-bp *pMYB98* fragment harboring *SaeM* in the middle is sufficient to drive SC-specific gene expression [1]. We discussed that it constitutes ANL2 and its homologs HDG1, HDG7, and EDT1 recognition motif at its 3′-end. Multiple potential ANL2 binding sites exist both downstream and upstream of the 84 bp promoter region. Notably, ANL2 and HDG1 harbor potential EAR-motifs (LxLxL) (see Adhikari, et al. [1]), recognized by TOPLESS (TPL) or TPL-related proteins (TPR) or SAP18, which interact with chromatin-associated histone deacetylase (e.g., HDA19) to confer their repressive effect [25,26]. Analysis revealed relatively low co-expression patterns of *ANL2* and *HDG1* with *MYB98* in FG component cells, suggesting their repressive role in *MYB98* expression. Preliminary analysis indicated that *ANL2* overexpression alone does not affect seed set—a proxy for normal *MYB98* expression and ovule function. Further investigation of shorter ANL2 splice variant, which constitutes an additional EAR motif near its C-terminus, as well as of *ANL2* homologs may offer clearer insights.

In addition to the ANL2-binding motif, the *SaeM* additionally harbors a potential GT-1 binding site (GT-element BOX III) at its 5′-end [1]. Other potential GT-1 binding sites downstream could be implicated in suppressing both overall and SC-specific reporter expression. GT elements, conserved across various plant species, reportedly determine tissue specificity of target genes by either activating or repressing gene expression depending on whether the GT factors are bound [17,31]. Our assessment indicates that GT-1, being non-SC-specific, might play a repressive role in *MYB98* expression. Confirmatory studies are needed to establish such a role of GT-1.

### 3.4. Limitations of Study

This study focuses on a workflow to analyze a pool of TFs potentially regulating the gene of interest (*MYB98*). TFs were identified using in vitro and in silico methods, with no direct validations from in vivo binding assays. Network analysis suggests potential physical associations of intermediary TFs, which were not identified in the Y1H assay. Wet lab experiments are necessary to confirm their interactions.

Genes such as *MYB63*, not directly linked to *MYB98* expression, may also exhibit expression differences between the *MYB98* domain and non-domain tissues. Such disparities may not necessarily imply a regulatory impact on *MYB98* expression.

Some genes participate in their own regulation through feedback loops. Apparently, *pMYB98* also constitutes a potential binding element (GTAACNT, [3]) for itself, yet the significance of this feedback in MYB98 regulation remains unclear. Since self-to-self expression correlation would always remain high irrespective of any kind of regulatory feedback loop, the current approach is insufficient to ascertain such an effect.

## 4. Materials and Methods

### 4.1. TF Pool Preparation

The initial pool of TFs was derived from the Y1H assay carried out during our earlier reported study [1]. Their putative binding sites were retrieved from the PlantPan 4.0 [19] and the CIS-BP database [20]. Additional TFs were added based on the DAP-seq data available at the Plant Cistrome Database [32].

### 4.2. Bioinformatics Analysis

The ATTED-II database (Arabidopsis) [19] was used for the co-expression analysis of the TFs; their network analysis was carried out using IntAct [24]; enrichment was assessed using eggNOG-mapper [33]; and potential master-regulators were identified using TF2Network [27].

## 5. Conclusions

Overall, the present study has developed a practical workflow to assign potential functions to a pool of TFs potentially regulating the expression of target gene (*MYB98*). Since *MYB98* is involved in conferring upon SCs the potential to attract PT, which is normally followed by SC burst; and since SC burst with no subsequent fertilization still leads to an increase in ovule size [34,35], a deeper understanding of its regulators could contribute to fertilization and apomixis research in the long run. The precision of current workflow-assisted TF functional role prediction could be greatly enhanced with access to extensive micro-tissue level or single-cell-specific expression data. The availability of multi-time-point expression data also promises to refine these predictions in a temporal dimension. For now, our predictions, based on limited datasets, at least serve as a preliminary guide for experimental design.

## Data Availability

All data associated with the study have been reported in this paper. Requests for additional relevant information will be cordially accepted by the authors.

## Supplementary Materials

The following supporting information can be downloaded at: https://www.mdpi.com/article/10.3390/plants13071007/s1, Figure S1: Regression and correlation assessments of putative *MYB98* expression regulating transcription factors (TFs). Each plot represents correlation/regression to individual TF. Plots are arranged from the highest (with positive regression) to lowest (with negative regression) R^2^-value. *MYB98* is plotted against itself in the very first plot as a positive control. (Data source: ATTED-II [19]). Figure S2: Network of TFs with potential physical associations to *pMYB98* binding members. Potential associations of some of the TFs with DNA and genes are shown. TFs derived from other species were excluded. Network was generated using IntAct. Supplementary Data S1. [Higher-resolution version of Figure 7].
