# Supplementary material for: Transcription Factors behind MYB98 Regulation: What Does the Discovery of SaeM Suggest?
Source: Plants (Basel). 2024 Mar 31;13(7):1007. doi: 10.3390/plants13071007 (PMC11013860; doi:10.3390/plants13071007)
Supplement: Supplementary file 1 [file plants-13-01007-s001.zip › plants-2871052-supplementary.pptx]

## Slide 1
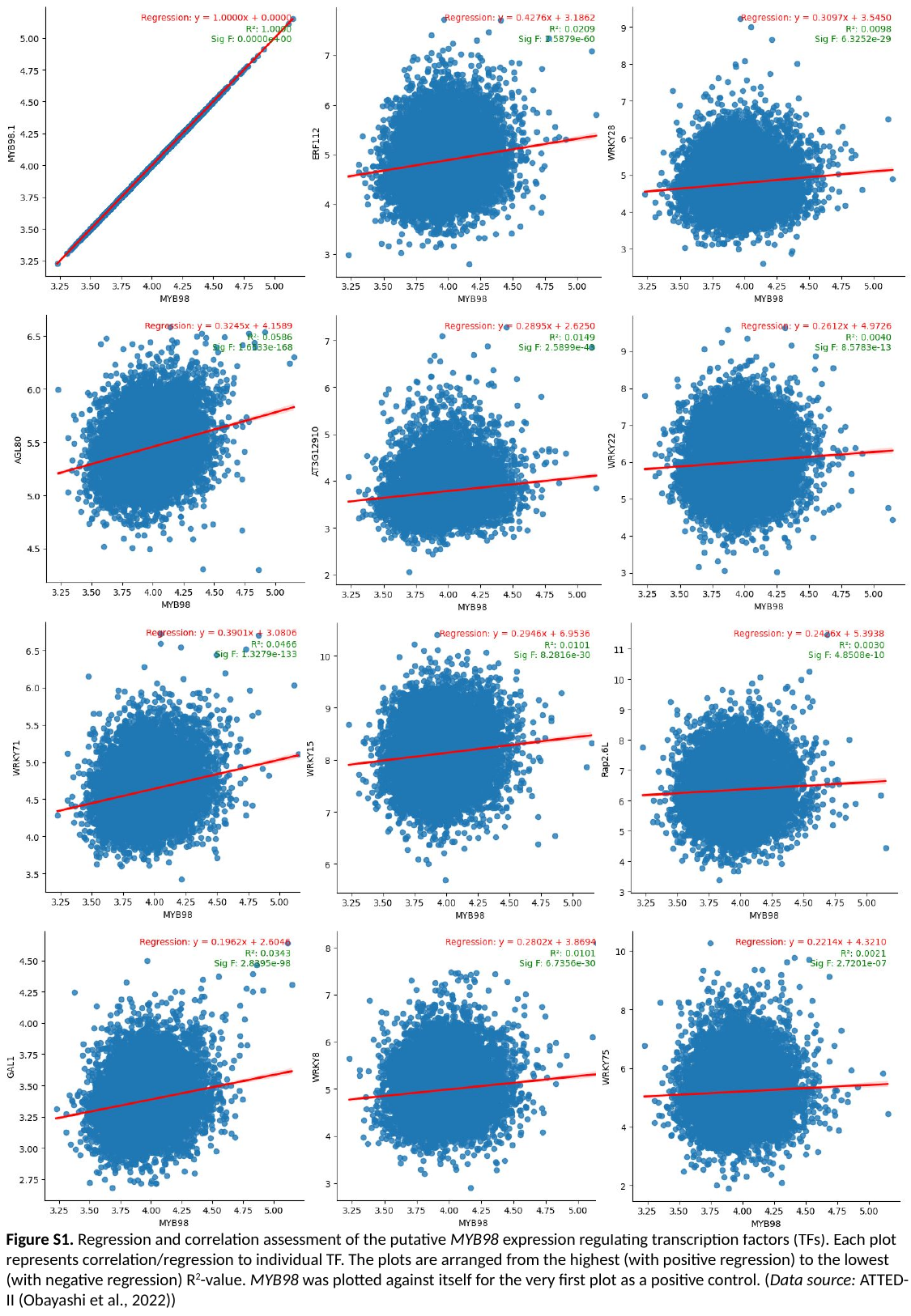

Figure S1. Regression and correlation assessment of the putative MYB98 expression regulating transcription factors (TFs). Each plot represents correlation/regression to individual TF. The plots are arranged from the highest (with positive regression) to the lowest (with negative regression) R2-value. MYB98 was plotted against itself for the very first plot as a positive control. (Data source: ATTED-II (Obayashi et al., 2022))

## Slide 2
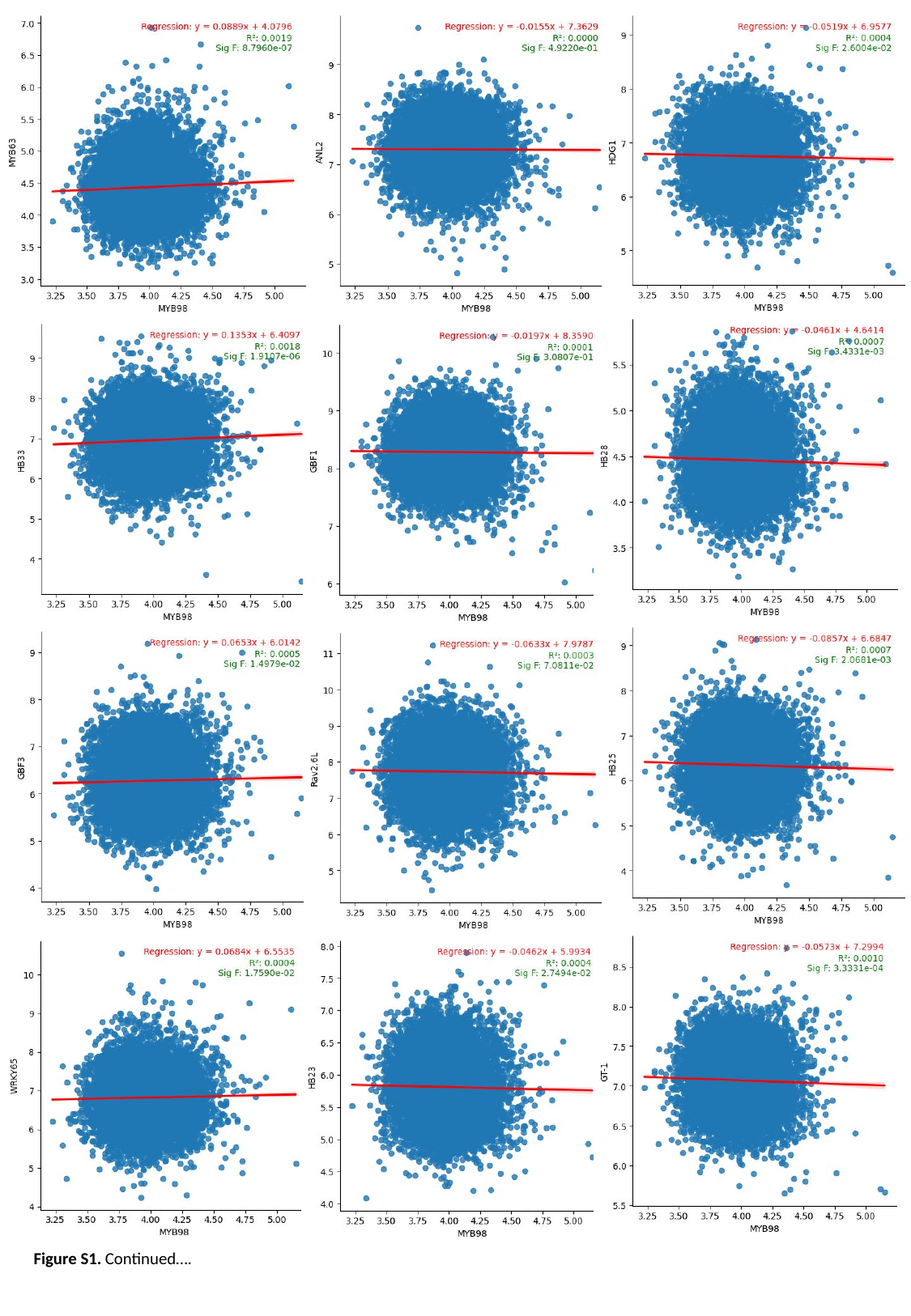

Figure S1. Continued….

## Slide 3
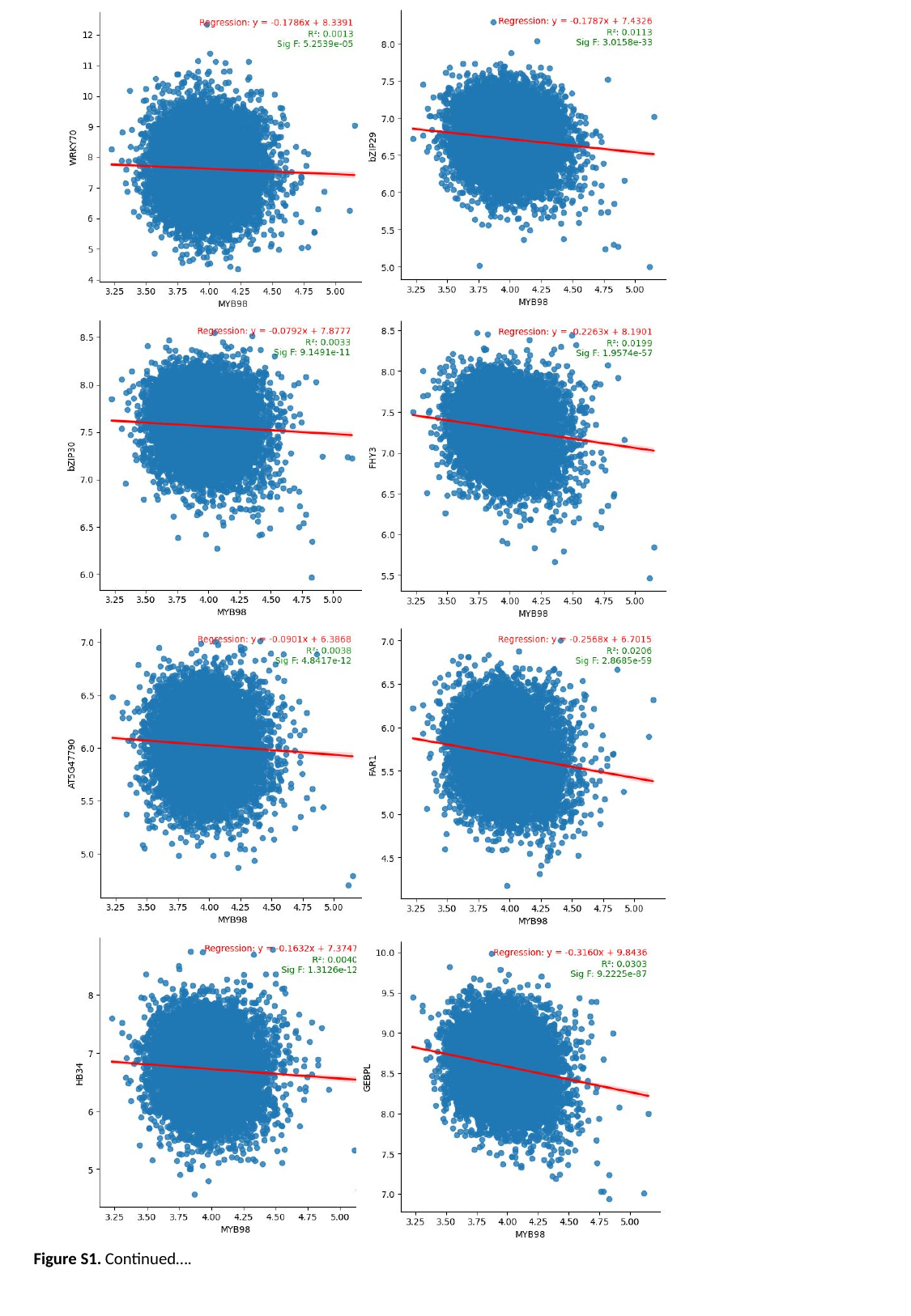

Figure S1. Continued….

## Slide 4
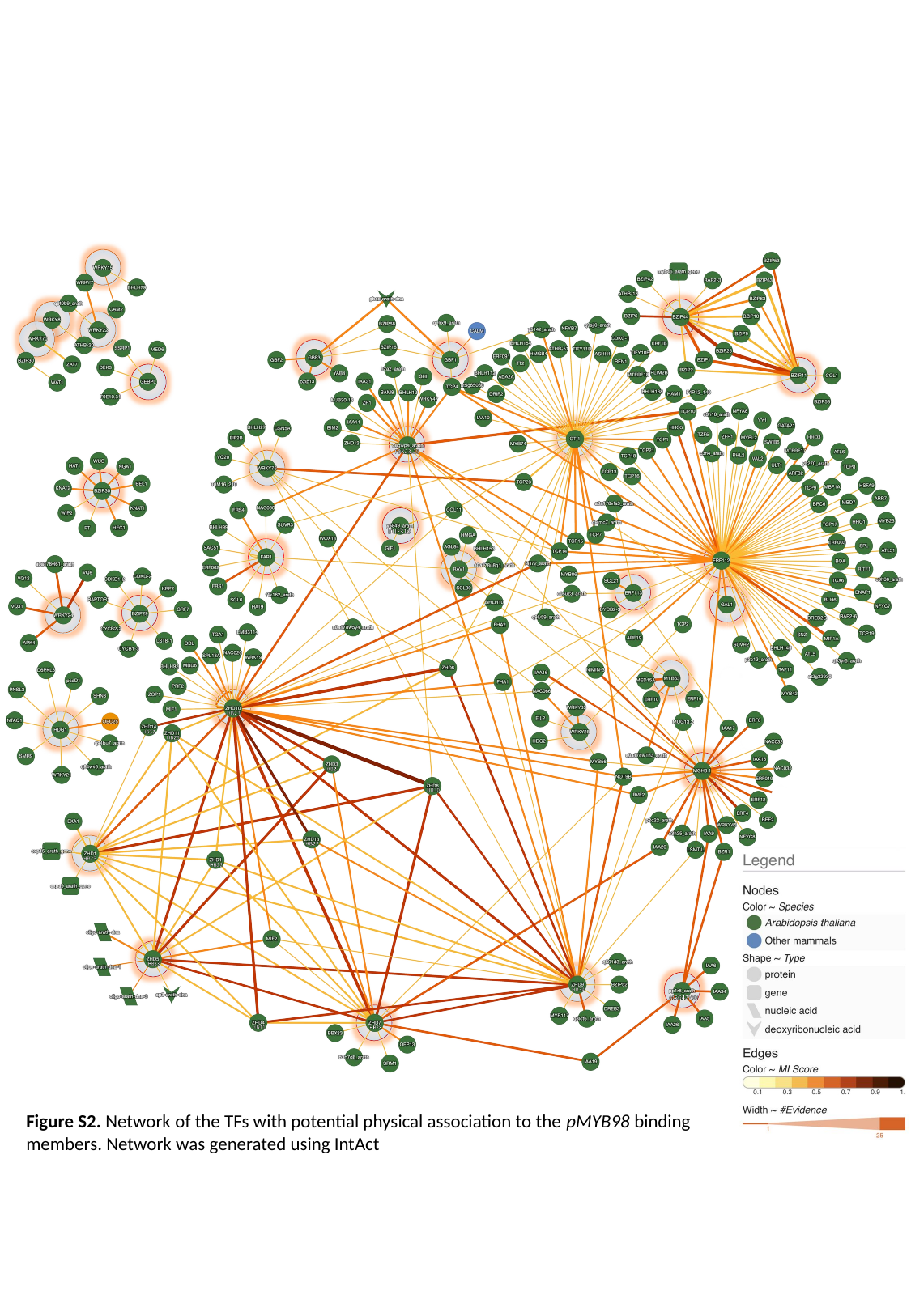

Figure S2. Network of the TFs with potential physical association to the pMYB98 binding members. Network was generated using IntAct

## Slide 5
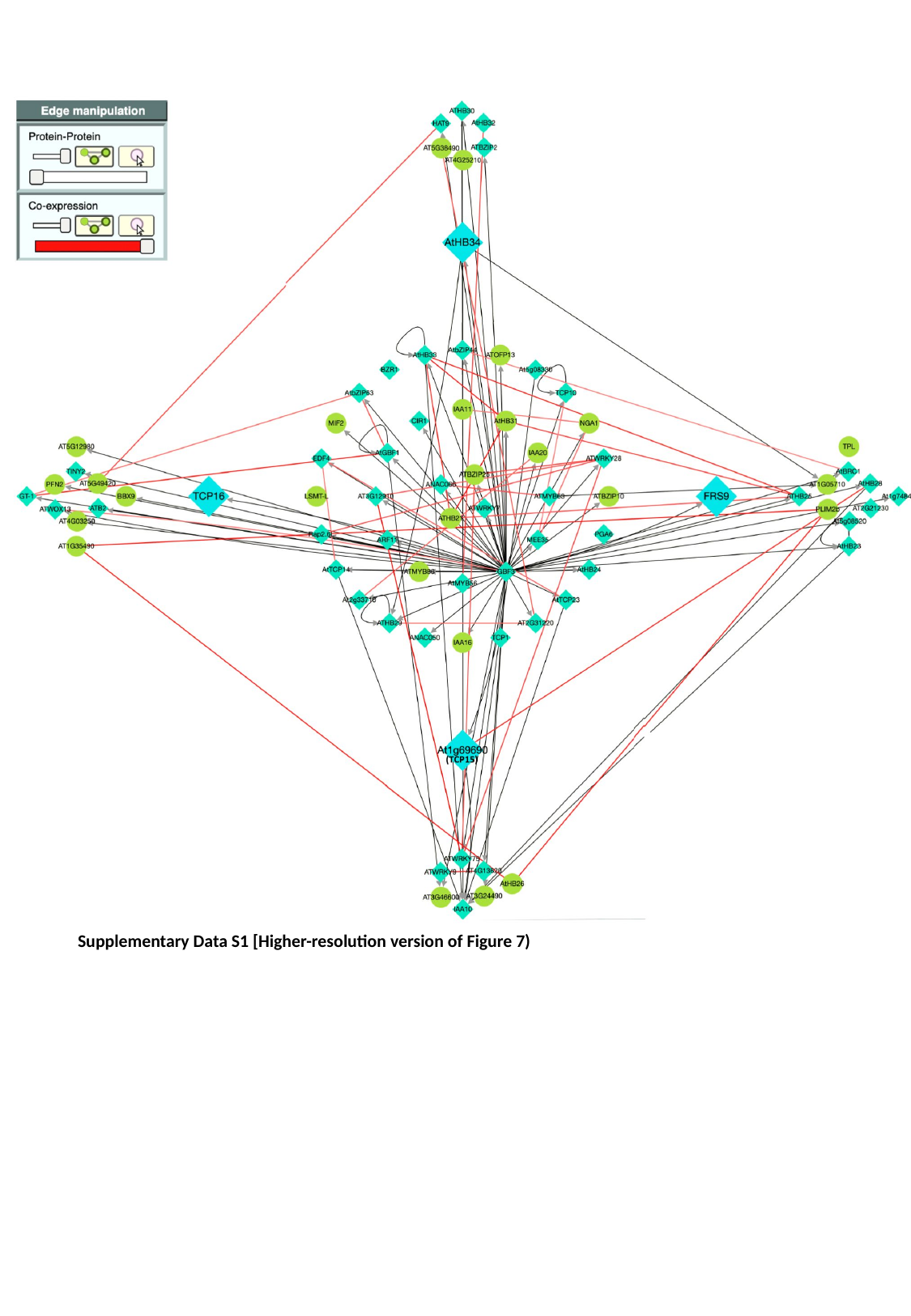

Supplementary Data S1 [Higher-resolution version of Figure 7)
